# Evaluation of nine HIV rapid test kits to develop a national HIV testing algorithm in Nigeria

**DOI:** 10.4102/ajlm.v4i1.224

**Published:** 2015-05-29

**Authors:** Orji Bassey, Kyle Bond, Adebayo Adedeji, Odafen Oke, Ado Abubakar, Kachiro Yakubu, Tapdiyel Jelpe, Ezekiel Akintunde, Patrick Ikani, Adeniyi Ogundiran, Ali Onoja, Issa Kawu, Gabriel Ikwulono, Idris Saliu, Okey Nwanyawu, Varough Deyde

**Affiliations:** 1 US Centers for Disease Control and Prevention (CDC), Nigeria; 2 Federal Ministry of Health (FMOH), Nigeria; 3 US Department of Defense (DOD), Nigeria; 4 Global HIV AIDS Initiative in Nigeria (GHAIN), Nigeria; 5 World Health Organization (WHO), Nigeria; 6 African Health Project (AHP), Nigeria; 7 Safe Blood for Africa Foundation (SBFAF), Nigeria

## Abstract

**Background:** Non-cold chain-dependent HIV rapid testing has been adopted in many resource-constrained nations as a strategy for reaching out to populations. HIV rapid test kits (RTKs) have the advantage of ease of use, low operational cost and short turnaround times. Before 2005, different RTKs had been used in Nigeria without formal evaluation. Between 2005 and 2007, a study was conducted to formally evaluate a number of RTKs and construct HIV testing algorithms.

**Objectives:** The objectives of this study were to assess and select HIV RTKs and develop national testing algorithms.

**Method:** Nine RTKs were evaluated using 528 well-characterised plasma samples. These comprised 198 HIV-positive specimens (37.5%) and 330 HIV-negative specimens (62.5%), collected nationally. Sensitivity and specificity were calculated with 95% confidence intervals for all nine RTKs singly and for serial and parallel combinations of six RTKs; and relative costs were estimated.

**Results:** Six of the nine RTKs met the selection criteria, including minimum sensitivity and specificity (both ≥ 99.0%) requirements. There were no significant differences in sensitivities or specificities of RTKs in the serial and parallel algorithms, but the cost of RTKs in parallel algorithms was twice that in serial algorithms. Consequently, three serial algorithms, comprising four test kits (Bundi^TM^, Determine^TM^, Stat-Pak^®^ and Uni-Gold^TM^) with 100.0% sensitivity and 99.1% – 100.0% specificity, were recommended and adopted as national interim testing algorithms in 2007.

**Conclusion:** This evaluation provides the first evidence for reliable combinations of RTKs for HIV testing in Nigeria. However, these RTKs need further evaluation in the field (Phase II) to re-validate their performance.

## Introduction

Nigeria is the tenth most populous country in the world and the most populous country in Africa, with an estimated population of 162.3 million.^1^ The first HIV case in Nigeria was reported in 1986.^2^ This stimulated interest in the screening of various populations in Nigeria for HIV.

The national HIV sero-prevalence sentinel survey amongst populations of pregnant women attending antenatal clinics (ANC) commenced in Nigeria in 1991 and has since become a biennial activity. The trend of HIV infection amongst this ANC population since the commencement showed a steady increase − 1.8% (1991), 3.8% (1993), 4.5% (1995, 1996), 5.4% (1999), to a high of 5.8% in 2001 – before declining to 5.0% in 2003 and then stabilising subsequently at 4.4% in 2005, 4.6% in 2008 and 4.1% in 2010.^3^ Nigeria has a generalised HIV epidemic – each of the 36 States and the Federal Capital Territory has over 1.0% HIV prevalence^4^ – and an estimated 3.5 million people are infected with the virus in the country. There are about 0.4 million estimated new infections per year, 1.5 million persons requiring antiretroviral therapy and an estimated 2.2 million total AIDS orphans currently living in the country.^5,6^ In 2005, the Nigeria National Action Committee on AIDS (NACA) strategic framework set out to provide antiretrovirals (ARVs) to 80.0% of adults and children with advanced HIV infection and to 80.0% of HIV-positive pregnant women, all by 2010. The implications of these efforts entail screening several million people for HIV infection. A 2005 survey of types of rapid test kits (RTKs) used in facilities participating in ANC in two of the six geopolitical zones of Nigeria revealed 19 different brands ranging from cold chain-dependent to non-cold chain-dependent (Adedeji AA, personal communication, March 2005). The lack of coordinated use of HIV RTKs also resulted in some discrepancies observed in results from the same sample within a health facility or at different health facilities, thereby making it difficult to provide centralised quality assurance or a post-marketing validation programme in-country (Adedeji AA, personal communication, March 2005). As a result of these problems, the Nigerian government saw the need to adopt the use of non-cold chain-dependent HIV RTKs for HIV testing.

HIV rapid testing remains a key entry point to HIV prevention, treatment, care and support in resource-limited settings.^7^ Its main advantages include the relative ease of use, low cost and faster turn-around time over enzyme immunoassays (EIAs) and Western blot (WB) assays. With an HIV rapid testing strategy, increased awareness of HIV status amongst many groups who would otherwise have been unaware of their status has been achieved.^8,9,10^ Providing quality-assured and accurate rapid HIV serological testing is critical in the early diagnosis and timely counselling of HIV-infected people for referral to care and treatment as well as prevention of mother-to-child transmission and monitoring of HIV prevalence in the population.^7,9^

HIV rapid testing also readily provides access to and enhances HIV counselling and testing in hard-to-reach rural populations,^11,12^ as well as in hard-to-reach, high-risk target populations, such as men who have sex with men.^10^ High-risk groups with acute HIV infection in Nigeria have previously been characterised by use of a combination of rapid HIV testing in mobile units and laboratory-based specimens pooling for nucleic acid amplification testing.^13^ To date, several African countries have conducted evaluation studies and implemented rapid HIV testing as a tool for fighting the HIV epidemic. These studies have demonstrated that the use of rapid testing can be an important part of the overall HIV testing strategies in resource-limited settings, where cold storage capacity, reliable power, efficient transportation and sufficient numbers of skilled laboratorians may not be readily available.^14,15,16,17,18,19,20^ A number of sub-Saharan African countries follow the World Health Organization (WHO) 2009 guidelines^21,22^ on the use of HIV antibody detection tests, where the recommended test algorithm includes a sensitive enzyme-linked immunosorbent assay (ELISA) as a screening test, followed by a confirmatory test done on all positive samples using WB.^23,24,25,26^ Recent studies have shown that diagnostic algorithms based on two or more serological tests are dependable and significantly lower the rate of recurrence of false positivity, thereby minimising misdiagnosis.^26,27^ Recently, the use of rapid testing combined with ELISA has increased significantly in Africa and Asia and tends to replace the use of WB assays.^26,28,29,30,31,32^ Accurate HIV diagnosis in resource-limited settings, as is the case in most regions of Nigeria, can be affected by emergence of new HIV subtypes and recombinant forms, hence the importance of occasionally assessing and selecting the best-performing serological assays before their wide-scale usage within the country.^26,33,34,35^

The goal of this evaluation was to assess and select non-cold chain-dependent HIV RTKs for the development of evidence based national testing algorithms based on key criteria such as performance, ease of use and cost, amongst others. It also sought to develop a list of highly-sensitive and specific HIV RTKs with documented good performance to serve as alternative algorithms in times of stock-outs of the RTKs included in the primary algorithms. The present evaluation allowed the identification and recommendation of three national interim algorithms for HIV rapid testing in the country. To our knowledge, these recommendations are still implemented by the Federal Ministry of Health (FMOH) and a second, field evaluation, phase has been conducted, although the results are not yet available. The methodology applied by the present evaluation could be used by other countries planning to develop HIV testing algorithms.

## Research method and design

### Strategy, sampling and testing

In August 2005, a multi-agency working group was set up by the government of Nigeria for the evaluation of HIV RTKs. The working group included participants from the FMOH and other organisations, specifically, the National AIDS and STIs Control Program (NASCP), NACA, the National Agency for Food, Drug Administration and Control (NAFDAC), the National Institute for Pharmaceutical Research and Development (NIPRD), the WHO, the Centers for Disease Control and Prevention – Global AIDS Program (CDC-GAP) and other partners implementing the US President's Emergency Plan for AIDS Relief (PEPFAR) programme in Nigeria, who had international experience in RTK evaluations.

### Test kit selection and characteristics

The HIV RTKs used in this evaluation were chosen based on the following WHO 2001 and 2009 recommended criteria: (1) stability within the climate in the country and not cold chain-dependent; (2) ability to test whole blood; (3) easy to use and interpret; (4) low test price (≤ US$3.20); (5) ability of manufacturers to produce and provide adequate numbers of testing kits to meet the needs of testing programmes in the country; (6) prior experience and validation – documented performance in the country and other African countries; (7) ability to detect HIV-1, HIV-2 and HIV type O subtypes; (8) ability to detect both IgG and IgM antibodies in order to reduce the window period; (9) do not require additional equipment to run tests or read results; (10) packaging of test kits not excessively bulky; (11) long shelf life (at least one year) and robust; and (12) test results provided in 30 minutes or less.^21,22^ In addition to the criteria above, test kits were selected based on their sensitivity and specificity when used singly and in combination using the minimum sensitivity and specificity (both ≥ 99.0%) criteria.^17,36^ The criteria were ranked in order of importance and relevance to the Nigerian context. A total of nine test kits were selected for the evaluation (Table 1). All the tests studied in this evaluation are qualitative tests for the detection of antibodies to HIV-1 and HIV-2 and use immunochromatographic technology.

**TABLE 1:** Characteristics of HIV rapid tests included in this evaluation.

Kit	Bundi^TM^ Rapid HIV 1/2	Determine^TM^ HIV-1/2	Double-Check Gold^TM^ HIV 1& 2	First Response® HIV 1-2.0	HIV 1/2 Stat-Pak® Assay	Insta-Chek HIV 1+2	OraQuick®Advance HIV-1/2	Sure-Check® HIV 1/2 (also called Clearview Complete)	Uni-Gold^TM^ HIV
Manufacturer	Bundi^TM^ International Diagnostics Ltd.	Abbott Laboratories	Orgenics, Ltd.	Premier Medical Corporation	Chembio Diagnostics Systems, Inc.	EY Laboratories	OraSure Technologies	Chembio Diagnostics Systems, Inc.	Trinity Biotech
Price per Test (US$)*	1.50	0.80	0.60	0.50	1.30	0.70	4.00	1.70	1.60
Tests per Kit	25	20 or 100	100	30	20	40 or 100	100	25	20
Cold-chain Dependent	No	No	No	No	No	No	No	No	No
Shelf-life (months)	12	14	12	15	18	18	6	12	15
Storage temperature (ºC)	4-30	2-30	2-30	4-30	8-30	-20-28	2-27	8-30	2-27
Sample type	Whole blood, serum, plasma	Whole blood, serum, plasma	Whole blood, serum, plasma	Whole blood, serum, plasma	Whole blood, serum, plasma	Whole blood, serum, plasma	Whole blood, serum, plasma, oral fluid	Whole blood, serum, plasma	Whole blood, serum, plasma
Sample volume required (µl)	10–20	50	10	10–20	5	40	5	2.5	60
Assay type	Immuno- chromatography	Immuno- chromatography	Immuno- chromatography	Immuno- chromatography	Immuno- chromatography	Immuno- chromatography	Immuno- chromatography	Immuno- chromatography	Immuno- chromatography
Solid phase	Membrane, Lateral flow	Membrane, Lateral flow	Membrane, Lateral flow	Membrane, Lateral flow	Membrane, Lateral flow	Membrane, Flow through	Membrane, Lateral flow	Membrane, Lateral flow	Membrane, Lateral flow
NAFDAC Registration Number	03-0653	03-0622	03-0875	03-0925	03-0934	Not Registered	Not Registered	03-0935	03-1011
USAID Procurement Approved	Yes	Yes	Yes	Yes	Yes	Yes	Yes	Yes	Yes
US Food and Drug Administration (FDA) approved	No	No	No	No	Yes	No	Yes	Yes	No

*, Costs shown in US dollars for the price in 2006; NAFDAC, National Agency for Food, Drug Administration and Control; USAID, United States Agency for International Development.

### Source and size of specimens

Specimens were collected from sites in five geopolitical zones of Nigeria between 2005 and 2006. Ten health facilities were originally planned to contribute specimens for this evaluation; however, because of logistical challenges, specimens from only five facilities were used for the study. These sites still provide a good representation of the population. Patient identification information was removed from all specimens and only HIV sero-status was reported. All specimens included in this study were unlinked and anonymised before inclusion and no blood specimen was drawn solely for the purpose of this validation.

The specimen panel used for this evaluation was prepared from two sources. The first was existing sample archives (leftover plasma or serum collected routinely for diagnostic purposes) in HIV testing laboratories at federally administrated teaching hospitals. The second was the remaining samples from a joint CDC/University of Maryland HIV sero-conversion project. The following specimen acceptance or rejection criteria were put in place to ensure that specimens of high quality were used in this evaluation: (1) properly collected, no haemolysis; (2) properly processed, no obvious signs of fungal or bacterial contamination/growth; (3) properly stored, freshly collected, at 20 °C, not stored for longer than two months at the collection sites; (4) clear HIV EIA sero-status, positive or negative. HIV-positive specimens had to contain high titres of HIV-specific antibody and an EIA signal-to-cutoff ratio of 3.0 or higher. HIV-negative specimens had to have EIA results comparable to that of the kit negative control; and (5) adequate specimen volume (at least 3.0 mL).

All specimens were treated and prepared based on the CDC/WHO guidelines.^15,16^ Specimens were then given new identification numbers, logged into a database and divided into about three aliquots (volume permitting) to avoid repeated freeze-thaw cycles that may affect antibody titres. The aliquots were stored at -20 **^°^**C for a maximum of two months until being characterised and used in the evaluation. To avoid several freeze-thaw cycles, aliquots were kept in a refrigerator whilst in use during the validation period.

Approximately 200 HIV-positive and 200 HIV-negative specimens are needed to provide 95% confidence intervals of less than ± 2.0% for both the estimated sensitivity and specificity. Thus, to meet the minimum acceptable test characteristics of the HIV rapid test as stated above, the final panel sample contained 528 specimens, of which 198 (37.5%) were HIV-1 positive and 330 (62.5%) were HIV negative.

### Testing procedure

All specimens were assigned new identification numbers between 1 and 528, then ordered by their reactivity (positive or negative) and randomised to allow for blinded testing. Ten skilled and experienced laboratorians working on the serology bench at the sites that contributed the specimens for the evaluation were recruited and were then provided with background information on the evaluation, refresher training on Good Laboratory Practice and an orientation to the data entry forms. Job aids were provided for each RTK and each test was demonstrated. Under the supervision of CDC and NASCP laboratory staff, the laboratorians practised on control specimens prior to evaluating the panel. Testing of each assay was implemented using the specimens according to the manufacturer's instructions for each individual test kit. Laboratorians worked in pairs; each pair evaluated approximately 100 specimens over a half-day period per test product. Specimen sets were rotated between the testers. Each test result was read independently by two individuals. All the laboratorians then completed a questionnaire concerning various aspects of the RTK they had just evaluated (see Appendices). The laboratorians appraised each RTK based on the following criteria: ease of running and reading test results, including ease of reading the reaction line; ease of interpreting the test results; ease of learning the test procedure; overall ease of running the assay; packaging size; and waste generation. This was done in an effort to capture information, in addition to accuracy, which is also critical in identifying tests for an algorithm.

### Reference testing/Gold standard

All specimens were fully characterised using standardised reference testing (gold standard): two third-generation EIAs, plus WB for all EIA-reactive specimens. All specimens with discordant EIA and WB results were excluded from the panel. Specimens with indeterminate WB results were also excluded.

The two EIAs selected for this validation, namely, Vironostika^®^ HIV Uniform II Plus O (Biomerieux, France) and Genscreen^®^ 1/2 Version 2 (Bio-Rad, USA), were both third-generation EIAs targeting both IgG and IgM of HIV-1 and -2, plus type O antibodies using recombinant antigens covering all group M, HIV-1 subtypes A-H. Both assays have been widely used throughout Africa^12,18,19,25^ and have consistently produced reliable data and detected HIV-specific antibodies. An antibody-only test is the most appropriate for comparison with HIV antibody-detecting rapid tests. The WB kit selected was New LAV-BLOT I (Bio-Rad). All reference testing was conducted as per the manufacturer's instructions.

### Quality control reference laboratory testing

All laboratory work associated with this evaluation was carried out at the Asokoro Training Laboratory, located at the Asokoro General Hospital in Abuja. This work included specimen characterisation, storage and the evaluation exercise. This Institute of Human Virology, Nigeria (IHVN)-supported site was selected for the following reasons: current status as a national HIV laboratory training facility; central location within Federal Capital Territory; constant electrical power; ongoing external quality assurance/laboratory monitoring programme; appropriate infrastructure for reference testing (EIA equipment); and adequate specimen storage space.

### Data collection, management and analysis

All test results were collected on paper forms and entered into a spreadsheet database (Microsoft^®^ Excel™) for analysis. Access to the project databases was limited to only key project staff through password-protected computers and all paper forms were kept in locked filing cabinets. During the data analysis, the sensitivity and specificity of each RTK were calculated by comparing the RTK results with reference results derived from EIA/WB testing.

### Cost estimations

The Supply Chain Management System (SCMS) was established in Nigeria in 2007 following the WHO HIV Test Kit Bulk Procurement Scheme established in 1989, which is aimed at facilitating access to high-quality test kits at a low cost through an easy purchasing procedure. The SCMS coordinates pooled procurement systems for HIV ARVs and RTKs and provided pricing information for the analysis as negotiated with the manufacturers and/or companies or their agents.^37,38^

Each of the RTKs under consideration was evaluated in both parallel and serial testing algorithms and anticipated costs for each algorithm were determined in US dollars based on the negotiated SCMS price. For the parallel testing algorithms, the price of each screening RTK was added to that of the confirmatory (i.e., second) RTK. The cost of the tie-breaker RTK was not included, since the frequency of use of a tie breaker is low (at most, 1.8% of the time). For the serial algorithms, the full price of the screening RTK was added to the price of the confirmatory RTK at 10.0% HIV prevalence (since the second test would only be used to confirm positive test results), plus the price of the tie breaker when needed at an HIV prevalence of 10.0%.

### Ethical considerations

The protocol for this evaluation was developed following the WHO's Regional Office for Africa (WHO AFRO) guidelines^21^ and received ethical approval from the NIPRD, Nigeria and Institutional Review Board (IRB) as well as the CDC IRB (approval dated 03.21.2006).

## Results

### Sensitivity and specificity of the evaluated individual test kits

The sensitivity and specificity results were calculated for each individual test (Table 2). All nine tests performed well in this evaluation, as indicated by high sensitivity and specificity values. The sensitivity value for seven of the nine tests was 100.0%, indicating that none of these tests produced false-negative results. Two tests, First Response^®^ and InstantChek^TM^, had lower sensitivities (98.9% and 96.9%, respectively). Specificity varied slightly between the tests, ranging from 96.0% to 100.0%; OraQuick^®^ and Stat-Pak^®^ were each 100.0% specific.

**TABLE 2:** Sensitivity and specificity calculations for the nine selected rapid test kits.

Test Kit Name	Results	Gold standard		Total	Sensitivity (95% Confidence Interval)^†^	Specificity (95% Confidence Interval)^‡^
		True Positive	True Negative			
Bundi^TM^	Positive	198	1	199	100.0% (98.1% – 100.0%)	99.7% (98.3% – 99.9%)
	Negative	0	329	329		
Determine^TM^	Positive	198	7	205	100.0% (98.1% – 100.0%)	97.8% (95.7% – 99.1%)
	Negative	0	323	323		
Double-Check Gold^TM^	Positive	198	7	205	100.0% (98.1% – 100.0%)	97.8% (95.7% – 99.1%)
	Negative	0	323	323		
First Response^®^	Positive	196	5	201	98.9% (94.4% – 99.8%)	98.4% (96.5% – 99.5%)
	Negative	2	325	327		
InstantChek^TM^	Positive	192	13	205	98.9% (96.3% – 99.8%)	96.1% (93.5% – 97.9%)
	Negative	6	317	323		
OraQuick^®^	Positive	198	0	198	100.0% (98.1% – 100.0%)	100.0% (98.8% – 100.0%)
	Negative	0	330	330		
Stat-Pak^®^	Positive	198	0	198	100.0% (98.1% – 100.0%)	100.0% (98.8% – 100.0%)
	Negative	0	330	330		
Sure-Check^®^	Positive	198	1	199	100.0% (98.1% – 100.0%)	99.7% (98.3% – 99.9%)
	Negative	0	329	329		
Uni-Gold^TM^	Positive	198	1	199	100.0% (98.1% – 100.0%)	99.7% (98.3% – 99.9%)
	Negative	0	329	329		
Total by Gold Standard Method^§^	-	198	330	-	-	-

RTK, rapid test kit; †, Number of true positives by RTK ÷ total true positives by the gold standard method x 100; ‡, Number of true negatives by RTK ÷ total true negative by the gold standard method x 100; §, Gold standard refers to two third-generation enzyme immunoassays (EIAs), plus Western blot for all EIA-reactive specimens.

### Kits dropped from consideration

**FIGURE 1:**
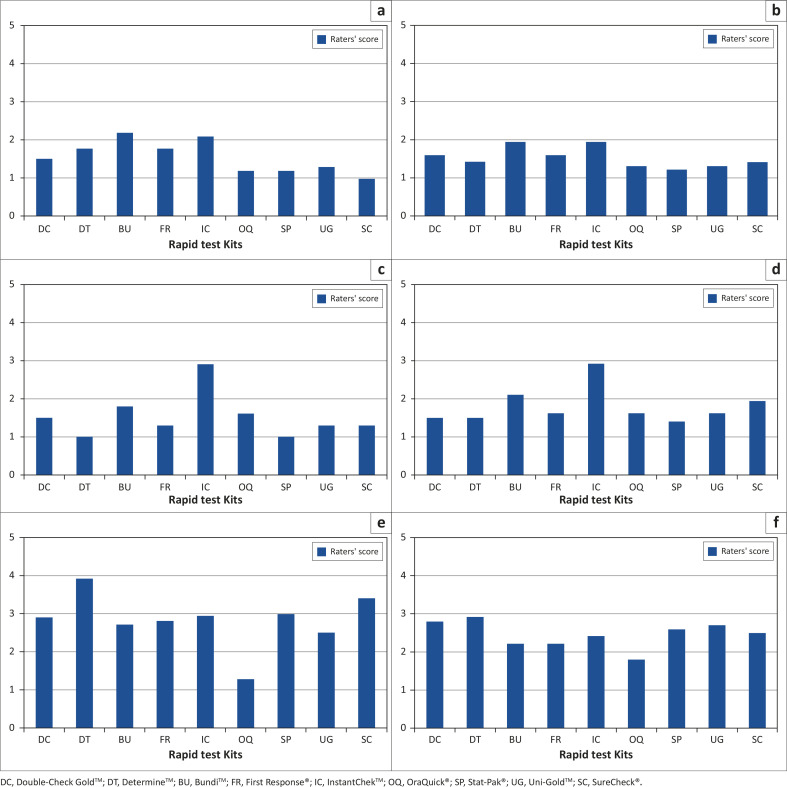
Process for selecting HIV rapid test kits for development of interim national HIV testing algorithms. Commercial kits available in Nigeria were selected for evaluation singly based on WHO guidelines (Step 1). Of the nine kits, six were retained for inclusion in the algorithm testing exercise and three were dropped (Step 2). Serial and parallel testing algorithms were assessed for performance (sensitivity and specificity), cost and the country context (Step 3). Serial algorithms using four kits were selected and recommended as interim national guidelines in 2007 (Step 4).

Figure 1 shows the kit selection process and results. After the initial performance of each individual kit was tested, three of the nine kits (InstantChek^TM^, First Response^®^ and OraQuick^®^) were removed from further consideration. Both InstantChek^TM^ and First Response^®^ ere excluded because of their performance (sensitivity and specificity) and the complexity of the result interpretation. OraQuick^®^ was dropped because of its cost and short shelf-life.

**FIGURE 2:**
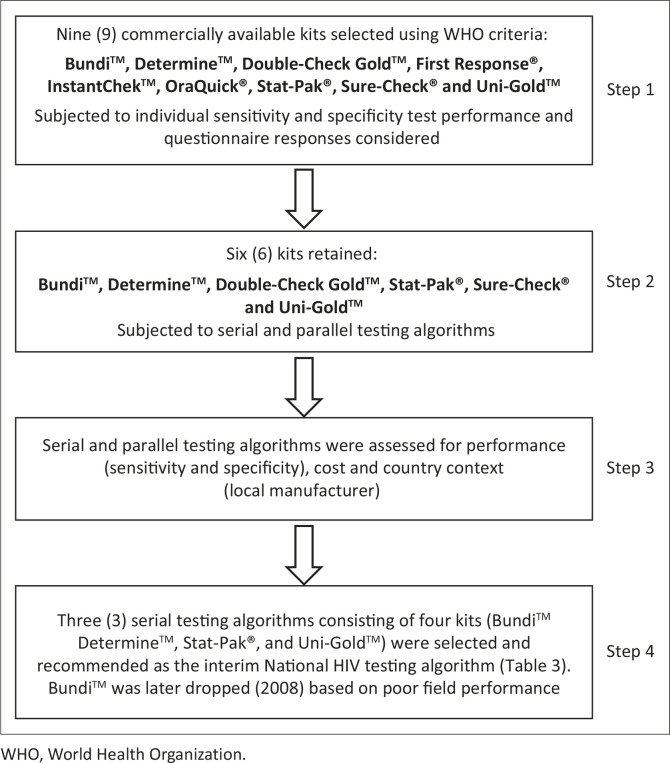
Results from questionnaires administered to laboratorians conducting the evaluation. Respondents were asked to rate all nine kits based on the following criteria: ease of reading the reaction line (panel a); ease of interpreting the test results (panel b); ease of learning how to perform the test (panel c); and overall ease of using and running the kit (panel d). Scores ranged from very easy (1) to very difficult (5) for this set of four questions; panels a–d represent average scores. Respondents were also asked about the size of the packaging (panel e), with scores ranging from 1 (very bulky) to 5 (very compact); and about quantity of waste generated (panel f), with scores ranging from 1 (a lot of waste) to 5 (minimal waste). Panels e-f represent average scores.

Figure 2 presents a summary of findings from the questionnaire on RTK characteristics administered to the laboratorians who performed the testing.

### Accuracy of testing algorithms

In diagnostic settings, RTKs are used in testing algorithms, not as individual tests. One major advantage of evaluating RTKs using a single, well-characterised specimen panel is that sensitivity and specificity can be calculated for all possible combinations of tests. This was completed for both parallel and serial algorithms (Box 1, Appendix 3 and 4).

**BOX 1:** Cost (US $), sensitivity and specificity of parallel and serial testing algorithms†.

Test Kit Combinations			Parallel Testing			Serial Testing		
Screening‡	Confirmatory‡	Tie breaker	Sensitivity	Specificity	Cost ($)^§^	Sensitivity	Specificity	Cost ($)^§^
Determine^TM^	Stat-Pak^®^	Bundi^TM^	100.0	99.7	2.20	100.0	99.7	0.99
Determine^TM^	Uni-Gold^TM^	Stat-Pak®	100.0	99.7	2.45	100.0	99.7	1.01
Determine^TM^	SureCheck®	Bundi^TM^	100.0	99.7	2.60	100.0	99.7	1.03
Determine^TM^	Uni-Gold^TM^	Bundi^TM^	100.0	99.4	2.45	100.0	99.4	1.01
Bundi^TM^	Determine^TM^	Double-Check Gold	100.0	99.4	2.35	100.0	99.7	1.59
Bundi^TM^	Double-Check Gold	Determine^TM^	100.0	99.7	2.14	100.0	99.7	1.66
Bundi^TM^	Stat-Pak®	Determine^TM^	100.0	99.7	2.85	100.0	99.7	1.64
SureCheck®	Uni-Gold^TM^	Bundi^TM^	100.0	100.0	3.35	100.0	100.0	1.91
Double-Check Gold^TM^	SureCheck®	Bundi^TM^	100.0	99.7	2.20	100.0	99.7	0.80
Stat-Pak®	SureCheck®	Bundi^TM^	100.0	100.0	3.10	100.0	100.0	1.53

†, The combinations represented here were selected based on the test kits chosen for the national testing algorithms for Nigeria; ‡, For the serial testing algorithms; §, The cost refers to the entire algorithm.

All possible parallel algorithms had a sensitivity of 100.0%, which indicates that none of the specimens in this panel had a false-negative result with more than one test product (Appendix 3). Specificity was also high, ranging from 99.1% to 100.0%. This represents from zero to three false-positive results for each algorithm. Over one-third (*n* = 24/60) of the possible test combinations had the highest possible (100.0%) sensitivity and specificity.

For all 120 possible serial algorithms, sensitivity was 100.0% (Appendix 4). Specificity ranged from 99.1% to 100.0%. Over half (*n* = 67) of the proposed algorithms had 100.0% sensitivity and specificity. This included five of the eight algorithms utilising the two RTKs currently in wide use in Nigeria (Determine^TM^ and Stat-Pak^®^).

The number of specimens (out of 528) requiring a tie-breaker test because of discordant results between the first two tests is also reported for serial and parallel algorithms (Appendix 3 and 4). Eight of 30 serial algorithms (two test combinations) did not require the use of a tie-breaker test. Other combinations, for both algorithm types, ranged from one to 10, representing at most 2.0% of specimens.

### Cost estimates for the algorithms

The cost for the serial testing algorithms was found to range from US$0.70 to US$1.90, whilst parallel algorithms ranged from US$1.40 to US$3.30 (Table 3, Appendix 3 and 4). In general, the cost for the serial testing algorithms was about half the cost of the parallel testing algorithms, since the latter require two tests to be run on all clients, even the 90.0% of clients who are HIV negative.

**TABLE 3:** National interim serial HIV rapid testing algorithm implemented in 2007.

Screening Test	Confirmation of Positives	Tie-breaker
Determine^TM^	Stat-Pak^®^	Bundi^TMa^
Uni-Gold^TM^	Stat-Pak^®^	Bundi^TMa^
Determine^TM^	Uni-Gold^TM^	Stat-Pak^®^

Because of performance issues with the Bundi^TM^ rapid testing kit, these two algorithms were discontinued in 2008.

WHO, World Health Organization.

### Proposed test algorithms

Determine^TM^ and Stat-Pak^®^, both of which have been evaluated and used widely internationally, have also been used widely in the Nigerian HIV programme since the 2001 and 2006 ANC surveys, respectively. Tremendous investment has been made in training large numbers of laboratory staff, including the adaptation of the training package for both test kits for use in Nigeria. Determine^TM^ has also been used widely throughout Africa. In this evaluation, both tests had high sensitivity and specificity individually and in the serial algorithms. Determine^TM^, with its high sensitivity (100.0%), is strongest as a screening test and was recommended as the first test in any proposed algorithm. Determine^TM^ was not recommended as a confirmatory test because of its lower specificity (97.8%). Use as a tie-breaker was only recommended in the event that Stat-Pak^®^ is not available.

Uni-Gold^TM^ has also been used widely internationally and performed well in this evaluation. In light of the fact that larger numbers of tests will soon be available in Nigeria to support HIV diagnostic testing programmes, it was also included in the interim national HIV rapid testing algorithm. Based on its performance and the need for ongoing quality assurance, adequacy of supply of kits and the development of a track record, it was recommended that Bundi^TM^ be included as a tie-breaker test. This would allow for continued monitoring of this new product. Based on the above findings, the construction of the three interim serial testing algorithms was based on four of the six rapid HIV test kits, namely Determine^TM^, Stat-Pak^®^, Uni-Gold^TM^ and Bundi^TM^ (Table 3).

## Discussion

The expansion of HIV prevention and care services in resource-constrained settings comes with great challenges regarding how to maintain quality-assured and accurate HIV testing as the number of HIV testing facilities increases.^21^ In addition, there are challenges associated with the quest for alternative, less expensive and efficient rapid HIV testing strategies, devoid of the supplemental confirmatory testing using the expensive WB assay and capable of retaining a high level of sensitivity in the face of the divergent HIV-1 subtypes dominating most sub-Saharan African countries.^14,15,16,17,32,33^ This is even further complicated by the move to decentralise HIV testing by involving fewer skilled and experienced laboratory/non-laboratory personnel.^17,32^

This study evaluated nine HIV RTKs using double EIAs as the reference test and WB as a supplemental confirmatory test for EIA-concordant reactive specimens. This serves as a gold-standard testing method for this evaluation and is comparable to the methods adopted in similar studies.^29,32,39,40^ All of these studies were in line with the CDC/WHO AFRO guidelines^17^ for HIV testing technologies in Africa.

Of the nine RTKs selected for the evaluation, three (OraQuick^®^, InstantChek^TM^ and First Response^®^) were dropped because of short shelf-life, poor performance, cost or complexity following the WHO phase 1 HIV RTK evaluation criteria. The remaining six RTKs (Bundi^TM^, Determine^TM^, Double-Check Gold^TM^, Stat-Pak^®^, SureCheck^®^ and Uni-Gold^TM^) were then subjected to parallel and serial testing algorithms in several possible combinations, resulting in combinations with high levels of sensitivity and specificity, as well as a high accuracy for diagnosing HIV infection.

Sixty possible parallel algorithms had costs ≤ US$3.20, had a sensitivity of 100.0% – indicating that no-false negative results were obtained with the panel of specimens – and had specificities ranging from 99.1% – 100.0%. This represents zero to three false-positive results for each of the algorithms. It was also observed that over one-third (*n* = 24/60) of the possible test combinations had the highest possible (100.0%) sensitivity and specificity. Of note, the remaining 60 possible parallel combinations of the RTKs were not presented in this report because of their higher cost (> $3.20).

Similarly, of the 120 possible serial algorithms, sensitivity was 100%, whilst specificity ranged from 99.1% – 100.0%. Additional analysis revealed that over half (*n* = 67/120) of the possible serial algorithms had 100.0% sensitivity and specificity, indicating that none of the panel specimens showed a false-negative or false-positive result with more than one test product.

A comparative analysis of the performance characteristics between the parallel and serial testing algorithms revealed no differences in accuracy regarding individual performance in diagnosing HIV infection. Similar comparative analyses of performance of combinations of ELISAs and RTKs in parallel or serial testing algorithms have shown that these combinations can also produce accurate results for HIV infections diagnosis.^17,27^

However, a comparative cost analysis between the two testing strategies showed a substantial difference, as the cost of carrying out a parallel testing algorithm is twice as expensive as the cost of a serial testing algorithm. These findings are comparable to those previously reported.^14,17^

Besides sensitivity, specificity and the cost of the testing algorithms, other important factors were also considered before making a choice of assay for the national testing algorithm. Over the years, the FMOH, through its HIV/AIDS Division in its efforts to implement national programmes has also significantly invested in terms of training the laboratorians and non-laboratorians involved in HIV testing using the Stat-Pak^®^ and Determine^TM^ HIV RTKs. This shows that both test kits are both commercially- and readily available and had wide-scale use in Nigeria. Furthermore, based on laboratorians’ evaluation and rating of the nine rapid test kits using questionnaires and the test selection criteria recommended by the WHO, Determine^TM^ was identified as the most compact test kit, allowing for less expensive transport and generating the least waste, thereby alleviating concerns about biohazard waste disposal at testing sites, whilst Stat-Pak^®^ ranked high in terms of readability of the reaction line and result interpretation. Cost-wise, a serial testing algorithm comprising Determine^TM^ and Stat-Pak^®^ was found to be inexpensive, as it costs less than one US dollar. The cost of the serial testing algorithm is vital, considering the expected testing targets and the large size of the voluntary counselling and testing programme in Nigeria. Also rated highly was Uni-Gold^TM^, which is known to be used widely internationally. Bundi^TM^, on the other hand, was included in the serial algorithm as a tie-breaker, because it is assembled locally and readily available, in addition to its high performance in the evaluation. The ease and convenience of performing the assay were also considered as in previous, similar studies.^17^

Based on the above findings and criteria, Nigeria adopted the serial HIV testing algorithm as an interim national testing algorithm (Table 3). Similar considerations and decision strategies were also adopted in a comparable study conducted in 11 African countries.^17^

The six non-cold chain-dependent test kits (Bundi^TM^, Determine^TM^, Double-Check Gold^TM^, Stat-Pak^®^, SureCheck® and Uni-Gold^TM^) performed well in this laboratory-based evaluation, both as individual tests and in serial testing algorithms. Data from this initial evaluation suggest that any combination of these six RTKs would perform well in a three-test, serial algorithm and that the tests with the highest sensitivity, such as Determine^TM^ and Uni-Gold^TM^, should be used as the screening test, whereas those with highest specificity, such as Stat-Pak^®^, should be used for confirmation.

### Limitations of the study

The present evaluation is not without limitations. First of all, this evaluation was limited to stored frozen plasma specimens and oral fluid was not collected for evaluation. As a result, the comparative advantage of using test kits with oral fluid or fresh specimens could not be evaluated. Another limitation was the variability in performance of some of the test kits in the hands of different testing personnel; this phenomenon has also been observed previously.^14,15,16,17^ In addition, the sensitivity of these test kits/testing algorithms is not well established and may differ based on HIV-1 subtypes, given the great genetic diversity of HIV-1 in Africa.^17^ As a result, the WHO developed guidelines to help country-based evaluation and implementation of rapid HIV testing.^17^ Considering these limitations, a formal HIV test kit performance evaluation should be an ongoing process that starts before testing implementation and continues after testing processes have been implemented in the field. As a result, since this evaluation provided data on laboratory-based validation, the selected RTKs should be field tested (Phase II) in varying combinations before a final national testing algorithm is selected. Furthermore, it is critical to ensure that the HIV test algorithms currently in place and future ones be monitored continuously through a quality-assurance programme (Phase III) developed within Nigeria. This quality-assurance programme should have the capacity to rapidly identify and correct testing problems related to the selected test kits and use of those kits in algorithms. Finally, it is important to note that, at the time of preparing the present manuscript, field monitoring had revealed a performance issue with Bundi^TM^ and the kit was removed from the algorithm in 2008. Only three kits thus remain in use (Determine^TM^, Uni-Gold^TM^ and Stat-Pak^®^). The second phase (field-based evaluation) was conducted in 2012, however, the results are not yet available (Adedeji AA, personal communication, June 2012).

### Conclusion

Three HIV testing algorithms with high sensitivity, specificity and accuracy to diagnose HIV infections were identified and recommended for use as interim national algorithms. These HIV testing algorithms provide a cheaper and more efficient alternative to WB supplemental confirmatory testing. The results of this analysis showed further that serial testing algorithms are not only sensitive and specific, but also less expensive. Finally, the present evaluation provides the first evidence-based and reliable combination of HIV test kits in Nigeria. It is important that a field, ‘point-of-care testing’ evaluation is conducted and the findings used to inform future decisions on what test kits to use in the country for accurate HIV testing.

## Trustworthiness

The current report reflects the findings observed by the technical working group, those who performed the testing, as well as the team that analysed the data.

### Reliability

The results of the experiments presented in this report were obtained using specimens collected in Nigeria and these results have been confirmed using WHO-recommended gold standard testing procedures for evaluating HIV rapid test kits. However, the methods of the evaluation can be applied in other countries.

### Validity

The development and recommendation of an interim HIV rapid testing algorithm in Nigeria demonstrated the study's success in achieving its goal. Not only were the test kits evaluated based on gold standard methods and procedures, but also the outcome of the study were scientific evidence-based recommendations that allowed the government of Nigeria to make informed decisions on what kits to use in their HIV testing programmes.

## Appendix 3

**TABLE 1-A3:** Parallel algorithms.

Two tests run simultaneously		Tie breaker Test	Sensitivity	Specificity	Number of times Tie Breaker required	Cost (US$)†
Bundi	Double Check Gold	Determine	100.00	99.40	6	2.10
		Stat-Pak	100.00	99.70		
		SureCheck	100.00	99.70		
		Uni-Gold	100.00	99.70		
Bundi	Stat-Pak	Determine	100.00	99.70	1	2.80
		Double-Check Gold	100.00	99.70		
		SureCheck	100.00	100.00		
		Uni-Gold	100.00	100.00		
Bundi	SureCheck	Determine	100.00	99.70	2	3.20
		Double-Check Gold	100.00	99.70		
		Stat-Pak	100.00	100.00		
		Uni-Gold	100.00	100.00		
Bundi	Uni-Gold	Determine	100.00	99.40	2	3.10
		Double-Check Gold	100.00	99.70		
		Stat-Pak	100.00	100.00		
		SureCheck	100.00	100.00		
Bundi	Determine	Double-Check Gold	100.00	99.40	6	2.30
		Stat-Pak	100.00	99.70		
		SureCheck	100.00	99.70		
		Uni-Gold	100.00	99.40		
Determine	Double Check Gold	Bundi	100.00	99.40	10	1.40
		Stat-Pak	100.00	99.40		
		SureCheck	100.00	99.40		
		Uni-Gold	100.00	99.10		
Determine	Stat-Pak‡	Bundi	100.00	99.70	7	2.20
		SureCheck	100.00	100.00		
		Uni-Gold	100.00	99.70		
		Double-Check Gold	100.00	99.40		
Determine	SureCheck	Bundi	100.00	99.70	8	2.60
		Double-Check Gold	100.00	99.40		
		Stat-Pak	100.00	100.00		
		Uni-Gold	100.00	99.70		
Determine	Uni-Gold	Bundi	100.00	99.40	6	2.40
		Double-Check Gold	100.00	99.10		
		Stat-Pak	100.00	99.70		
		SureCheck	100.00	99.70		
Double-Check Gold	Stat-Pak	Bundi	100.00	99.70	7	1.90
		Determine	100.00	99.40		
		SureCheck	100.00	100.00		
		Uni-Gold	100.00	100.00		
Double-Check Gold	SureCheck	Bundi	100.00	99.70	8	2.30
		Determine	100.00	99.40		
		Stat-Pak	100.00	100.00		
		Uni-Gold	100.00	100.00		
Double-Check Gold	Uni-Gold	Bundi	100.00	99.70	8	2.20
		Determine	100.00	99.10		
		Stat-Pak	100.00	100.00		
		SureCheck	100.00	100.00		
Stat-Pak	SureCheck	Bundi	100.00	100.00	1	3.10
		Determine	100.00	100.00		
		Double-Check Gold	100.00	100.00		
		Uni-Gold	100.00	100.00		
Stat-Pak	Uni-Gold	Bundi	100.00	100.00	1	2.90
		Determine	100.00	99.70		
		Double-Check Gold	100.00	100.00		
		SureCheck	100.00	100.00		
SureCheck	Uni-Gold	Bundi	100.00	100.00	2	3.30
		Determine	100.00	99.70		
		Double-Check Gold	100.00	100.00		
		Stat-Pak	100.00	100.00		

Parallel algorithms: †, Assuming the tie breaker test is required about 1.0% of the time; ‡, These are the first two tests used in the current test algorithm in Nigeria.

## Appendix 4

**TABLE 1-A4:** Serial Algorithms.

First test	Second test	Tie breaker Test	Sensitivity	Specificity	Number of Times Tie Breaker required	Cost (US$)*
Determine	Bundi	Double-Check Gold	100.00	99.40	6	1.00
		Stat-Pak	100.00	99.70		
		SureCheck	100.00	99.70		
		Uni-Gold	100.00	99.40		
Bundi	Determine	Double-Check Gold	100.00	99.70	0	1.50
		Stat-Pak	100.00	99.70		
		SureCheck	100.00	99.70		
		Uni-Gold	100.00	99.70		
Determine	Double-Check Gold	Bundi	100.00	99.40	5q	0.90
		Stat-Pak	100.00	99.40		
		SureCheck	100.00	99.40		
		Uni-Gold	100.00	99.10		
Double-Check Gold	Determine	Bundi	100.00	99.40	5	0.70
		Stat-Pak	100.00	99.40		
		SureCheck	100.00	99.40		
		Uni-Gold	100.00	99.40		
Determine	Stat-Pak	Bundi	100.00	99.70	7	0.90
		Double-Check Gold	100.00	99.40		
		SureCheck	100.00	100.00		
		Uni-Gold	100.00	99.70		
Stat-Pak	Determine	Bundi	100.00	100.00	0	1.40
		Double-Check Gold	100.00	100.00		
		SureCheck	100.00	100.00		
		Uni-Gold	100.00	100.00		
Determine	SureCheck	Bundi	100.00	99.70	7	1.00
		Double-Check Gold	100.00	99.40		
		Stat-Pak	100.00	100.00		
		Uni-Gold	100.00	99.70		
SureCheck	Determine	Bundi	100.00	100.00	1	1.80
		Double-Check Gold	100.00	100.00		
		Stat-Pak	100.0	100.00		
		Uni-Gold	100.0	100.00		
Determine	Uni-Gold	Bundi	100.0	99.40	6	1.00
		Double-Check Gold	100.0	99.10		
		Stat-Pak	100.0	99.70		
		SureCheck	100.0	99.70		
Uni-Gold	Determine	Bundi	100.0	99.70	0	1.60
		Double-Check Gold	100.0	99.70		
		Stat-Pak	100.0	99.70		
		SureCheck	100.0	99.70		
Bundi	Double-Check Gold	Determine	100.0	99.70	0	1.60
		Stat-Pak	100.0	99.70		
		SureCheck	100.0	99.70		
		Uni-Gold	100.0	99.70		
Double-Check Gold	Bundi	Determine	100.0	99.40	6	0.70
		Stat-Pak	100.0	99.70		
		SureCheck	100.0	99.70		
		Uni-Gold	100.0	99.70		
Bundi	Stat-Pak	Determine	100.0	99.70	1	1.60
		Double-Check Gold	100.0	99.70		
		SureCheck	100.00	100.00		
		Uni-Gold	100.00	100.00		
Stat-Pak	Bundi	Determine	100.00	100.00	0	1.50
		Double-Check Gold	100.00	100.00		
		SureCheck	100.00	100.00		
		Uni-Gold	100.00	100.00		
Bundi	SureCheck	Determine	100.00	99.70	1	1.60
		Double-Check Gold	100.00	99.70		
		Stat-Pak	100.00	100.00		
		Uni-Gold	100.00	100.00		
SureCheck	Bundi	Determine	100.00	100.00	1	1.90
		Double-Check Gold	100.00	100.00		
		Stat-Pak	100.00	100.00		
		Uni-Gold	100.00	100.00		
Bundi	Uni-Gold	Determine	100.00	99.70	1	1.60
		Double-Check Gold	100.00	99.70		
		Stat-Pak	100.00	100.00		
		SureCheck	100.00	100.00		
Uni-Gold	Bundi	Determine	100.00	99.70	1	1.70
		Double-Check Gold	100.00	100.00		
		Stat-Pak	100.00	100.00		
		SureCheck	100.00	100.00		
Double-Check Gold	Stat-Pak	Determine	100.00	99.40	7	0.70
		Bundi	100.00	99.70		
		SureCheck	100.00	100.00		
		Uni-Gold	100.00	100.00		
Stat-Pak	Double-Check Gold	Determine	100.00	100.00	0	1.40
		Bundi	100.00	100.00		
		SureCheck	100.00	100.00		
		Uni-Gold	100.00	100.00		
Double-Check Gold	SureCheck	Determine	100.00	99.40	7	0.80
		Bundi	100.00	99.70		
		Stat-Pak	100.00	100.00		
		Uni-Gold	100.00	100.00		
SureCheck	Double-Check Gold	Determine	100.00	100.00	1	1.80
		Bundi	100.00	100.00		
		Stat-Pak	100.00	100.00		
		Uni-Gold	100.00	100.00		
Double-Check Gold	Uni-Gold	Determine	100.00	99.40	7	0.80
		Bundi	100.00	99.70		
		Stat-Pak	100.00	100.00		
		SureCheck	100.00	100.00		
Uni-Gold	Double-Check Gold	Determine	100.00	99.70	1	1.60
		Bundi	100.00	100.00		
		Stat-Pak	100.00	100.00		
		SureCheck	100.00	100.00		
Stat-Pak	SureCheck	Determine	100.00	100.00	0	1.50
		Bundi	100.00	100.00		
		Double-Check Gold	100.00	100.00		
		Uni-Gold	100.00	100.00		
SureCheck	Stat-Pak	Determine	100.00	100.00	1	1.80
		Bundi	100.00	100.00		
		Double-Check Gold	100.00	100.00		
		Uni-Gold	100.00	100.00		
Stat-Pak	Uni-Gold	Determine	100.00	100.00	0	1.50
		Bundi	100.00	100.00		
		Double-Check Gold	100.00	100.00		
		SureCheck	100.00	100.00		
Uni-Gold	Stat-Pak	Determine	100.00	99.70	1	1.70
		Bundi	100.00	100.00		
		Double-Check Gold	100.00	100.00		
		SureCheck	100.00	100.00		
SureCheck	Uni-Gold	Determine	100.00	100.00	1	1.90
		Bundi	100.00	100.00		
		Double-Check Gold	100.00	100.00		
		Stat-Pak	100.00	100.00		
Uni-Gold	SureCheck	Determine	100.00	100.00	1	1.70
		Bundi	100.00	100.00		
		Double-Check Gold	100.00	100.00		
		Stat-Pak	100.00	100.00		

Serial Algorithms: *, Assuming a prevalence of 10.0% attesting sites.
